# Functional Micropeptides Encoded by Long Non-Coding RNAs: A Comprehensive Review

**DOI:** 10.3389/fmolb.2022.817517

**Published:** 2022-06-13

**Authors:** Jianfeng Pan, Ruijun Wang, Fangzheng Shang, Rong Ma, Youjun Rong, Yanjun Zhang

**Affiliations:** ^1^ College of Animal Science, Inner Mongolia Agricultural University, Hohhot, China; ^2^ Key Laboratory of Mutton Sheep Genetics and Breeding, Ministry of Agriculture, Hohhot, China; ^3^ Key Laboratory of Animal Genetics, Breeding and Reproduction, Hohhot, China; ^4^ Engineering Research Center for Goat Genetics and Breeding, Hohhot, China

**Keywords:** lncRNA, micropeptide, sORF, Ribo-seq, coding potential prediction

## Abstract

Long non-coding RNAs (lncRNAs) were originally defined as non-coding RNAs (ncRNAs) which lack protein-coding ability. However, with the emergence of technologies such as ribosome profiling sequencing and ribosome-nascent chain complex sequencing, it has been demonstrated that most lncRNAs have short open reading frames hence the potential to encode functional micropeptides. Such micropeptides have been described to be widely involved in life-sustaining activities in several organisms, such as homeostasis regulation, disease, and tumor occurrence, and development, and morphological development of animals, and plants. In this review, we focus on the latest developments in the field of lncRNA-encoded micropeptides, and describe the relevant computational tools and techniques for micropeptide prediction and identification. This review aims to serve as a reference for future research studies on lncRNA-encoded micropeptides.

## 1 Introduction

Non-coding RNAs (ncRNAs) are generally considered as a class of RNAs that lack protein-coding ability. Based on their regulatory functions, ncRNAs can be categorized as long non-coding RNAs (lncRNAs), primary miRNAs (pri-miRNAs), circular RNAs (circRNAs), among others (Beermann et al., 2016; Khalili-Tanha and Moghbeli, 2021). LncRNAs have transcriptional length that exceeds 200 nucleotides, being initially defined as “transcriptional noise” (Choi et al., 2019). However, with the emergence and increasing use of high-throughput technologies such as ribosome profiling sequencing (Ribo-Seq) and ribosome-nascent chain complex sequencing (RNC-Seq), it has been demonstrated that lncRNAs have short open reading frames (sORFs) encoding micropeptides (Ruiz-Orera et al., 2020). However, the function of most encoded micropeptides has been overlooked due to their low molecular weight (100 amino acid residues or fewer).

LncRNAs are mainly transcribed by RNA polymerase II (Pol II) and have a structure similar to mRNA, including a 7-methylguanosine triphosphate (m7G-cap) at the 5' end and a poly(A) tail at the 3' end (Zhang et al., 2019; Statello et al., 2021), suggesting that lncRNAs may have a translational function comparable to that of mRNAs. However, unlike mRNAs, lncRNAs have distinct transcription, processing, and modification processes (Quinn and Chang, 2016). In addition, poor conservation and spatiotemporal specificity of lncRNAs expression greatly hinder the exploration of lncRNA coding potential (Nitsche and Stadler, 2017).

Previous studies have been demonstrated that, in addition to lncRNAs with coding potential, pri-miRNAs and circRNAs also possess sORFs encoding functional micropeptides. In this context, pri-miRNAs are a distinct type of lncRNAs of which the length is within the range of hundreds to thousands of nucleotides, being produced by Pol II. Thus, in this sense, pri-miRNAs may be similar to lncRNAs due to the micropeptide-encoding ability (Lauressergues et al., 2015; Lv et al., 2016; Wu P et al., 2020; Prasad et al., 2021). In contrast, circRNAs are transcribed by Pol II without the 5' cap and the 3' poly(A) tail, being thus resistant to digestion by RNaseR and having a ten-fold longer half-life compared to linear RNA (Lei et al., 2020). In addition, there is evidence that circRNAs possess highly conserved sORFs encoding functional micropeptides in a 5' cap-independent manner. Since circRNAs have a unique covalently closed structure sORFs therein circulate across the splicing site and even beyond their length (Shi et al., 2020; Wu S et al., 2020), and they can potentially encode micropeptides containing more than 100 amino acids in length. Collectively, these observations indicate that ncRNAs have potential applications in the field of encoding micropeptides which need to be further explored.

This review outlines the translational mechanisms of lncRNA-encoded micropeptides as well as the computational tools and techniques related to micropeptide prediction and identification. A discussion is also proposed on the latest research advancements of therapies based on lncRNA-encoded micropeptides, such as those applied to skeletal muscle, innate immunity, cancer, among others. Finally, it is summarized future outlooks on the current research landscape of lncRNA-encoded micropeptides, aiming to provide positive strategies, and novel insights for the future of micropeptide research.

## 2 Translational Mechanisms of lncRNA-Encoded Micropeptides

LncRNAs with coding ability have been described as early as in 2014. Ruiz-Orera et al. (2014) found that the majority of lncRNAs expressed in cells from six different species (human, mice, fish, flies, yeast, and plant) were linked to ribosomes. In addition, the ribosomal conservation pattern was consistent with the translation of micropeptides (Ruiz-Orera et al., 2014). Moreover, lncRNAs showed coding potential and structural constraints similar to those of nascent protein-coding sequences, suggesting that lncRNAs may play an important role in the *de novo* evolution of proteins (Ruiz-Orera et al., 2014). In 2014, Pauli et al. (2014) identified a conserved peptide encoded by an lncRNA, termed Toddler, involved in zebrafish embryogenesis. It has been demonstrated that both the lack and overexpression of this peptide reduced the movement of mesodermal cells during zebrafish gastrulation (Pauli et al., 2014). In a study by Chen et al. (2020), a strategy combining ribosome profiling, mass spectrometry (MS)-based proteomics, microscopy, and CRISPR-based genetic screening was used to explore and characterize widespread translation of functional micropeptides as well as determine the protein-coding potential of complex genomes. Using this screening strategy, hundreds of non-canonical lncRNA coding DNA sequences (CDSs) encoding stable functional micropeptides were identified as essential for cell growth and whose disruption triggered specific and robust transcriptomic and phenotypic changes in human cells (Chen et al., 2020). Thus, lncRNA-encoded micropeptides have been gaining increasing attention in research, being less considered a “translation noise” but rather functional micropeptides.

In 2015, Ji et al. (2015) identified that 40% of lncRNAs and pseudogene RNAs expressed in human cells are translated. In addition, these authors verified that approximately 35% of mRNA-encoding genes are translated upstream of primary protein-coding regions (uORFs), and 4% are translated downstream (dORFs) (Ji et al., 2015). In this same study, it has been demonstrated that translated lncRNAs are preferentially localized in the cytoplasm, while non-translated lncRNAs are preferentially found in the nucleus (Ji et al., 2015). Translation efficiency of cytoplasmic lncRNAs was shown to be comparable to that of mRNAs, indicating that sORFs of cytoplasmic lncRNAs are protected by ribosomes and involved in translation (Ji et al., 2015). Common ORFs are defined as the DNA sequence found between the start (ATG or AUG) and stop codons (TAG or TGA) (Sieber et al., 2018), whereas sORFs typically possess less than 300 nucleotides in length, and longer sORFs are more likely to be translated (Pueyo et al., 2016; Orr et al., 2020). It has also been found that regulatory elements upstream of ORFs, e.g., internal ribosome entry site (IRES), N6-methyladenosine (m6A) methylation conserved sites, can mediate micropeptide translation (Wu P et al., 2020; Charpentier et al., 2022). IRES elements are important regulatory RNA sequences that do not rely on 5' cap for translation, which mostly occur in the 5' untranslated region (5' UTR) upstream of the ORF controlled by IRES (Zhao et al., 2018). By recruiting ribosomes and then proceeding to ribosome assembly, translation of sORFs into micropeptides can occur. In addition, IRES elements may also be present between and within ORFs to mediate translation, and lncRNAs with IRES elements can be translated into micropeptides based on consecutive sORFs (Stoneley and Willis, 2004; King et al., 2010; Hanson et al., 2012; Carbonnelle et al., 2013). Furthermore, it has been demonstrated that m6A can drive endogenous ncRNA translation, in particular the translation of circRNA, and hundreds of endogenous circRNA with translation potential have been identified (Yang et al., 2017), which greatly enlarges our study. Moreover, it can be speculated that m6A could also potentially drive endogenous lncRNA translation.

The translational capacity of lncRNAs is regulated by proteins in addition to post-transcriptional regulation mechanisms (e.g., splicing, polyadenylation). The micropeptide STORM encoded by linc00689 is regulated by phosphorylation of the eukaryotic translation initiation factor 4E (eIF4E) which is mediated by TNF-α and mammalian Ste20-like kinase (MST1) (Min et al., 2017). eIF4E is an mRNA cap-binding protein that is a general initiation factor allowing for mRNA-ribosome interaction and cap-dependent translation in eukaryotic cells (Ross-Kaschitza and Altmann, 2020). Phosphorylation of eIF4E was found to weaken the interaction with 5' cap while inhibiting mRNA translation, but enhanced the association of active polyribosomes with lncRNA (Min et al., 2017).

Nonsense-mediated decay (NMD) is an important mechanism for mRNA quality monitoring. NMD is triggered by long 3' UTR, and intronless genes may be insensitive to NMD (Tan et al., 2021). Wery et al. (2016) , using ribosomal analysis, described that actively translated lncRNA sORFs with long 3' UTR were responsive to NMD, suggesting that NMD may also be a monitoring mechanism for lncRNA translation. In addition, it has been suggested that micropeptides encoded by lncRNAs interact with the mRNA decapping protein complex which is responsible for the removal of the 5' cap from mRNA to promote 5' to 3' decay (DLima et al., 2017). Simultaneously, micropeptides encoded by lncRNAs can also be co-localized with mRNA decay-associated RNA protein granules to alter the steady-state levels of cellular NMD targets (D'Lima et al., 2017). Collectively, the above results illustrate that lncRNAs have mRNA-like translational functions of which mechanisms are regulated by a variety of regulatory proteins as well as by NMD monitoring. In addition, micropeptides encoded by lncRNAs have been shown to regulate NMD homeostasis. These findings suggest that micropeptides have a promising regulatory role, which requires further studies in order to elucidate currently unknown regulatory mechanisms.

## 3 Prediction and Identification of lncRNAs Coding Ability

### 3.1 Sequencing Analysis Based on “Omics” Techniques

Most of current studies on lncRNA-encoded micropeptides are based on data obtained by ribosome analysis (Ruiz-Orera and Alba, 2019). However, “omics” techniques have been considered an important tool to study the coding capacity of lncRNAs. In this context, translational omics analysis has been commonly used, and mainly relies on four techniques (Ingolia et al., 2009; Ingolia et al., 2019; Zhao et al., 2019): polysome profiling, ribosome-nascent chain complex sequencing (RNC-Seq), ribosome affinity purification (TRAP-Seq), and ribosome profiling (Ribo-Seq) (Table 1).

**TABLE 1:** Advantages and disadvantages of translation-nomics related techniques.

Techniques	Advantages	Disadvantages	References
Polysome profiling	RNC-mRNA can be obtained; any length, sequence variation, number of ribosomes on each mRNA can be detected	It is difficult to perform in-depth analysis of all translated mRNA	Chasse et al. (2017)
RNC-Seq	It can effectively reveal the full-length information of the RNA being translated, including abundance, and type	Prone to ribosome dissociation or RNA degradation after cell lysis; low sequencing precision; no access to ribosome, ORF, uORF information	Wang L et al. (2013)
TRAP-Seq	RNC-mRNA can be obtained; avoids contamination by eliminating the need for ultracentrifugation; it has the advantage of isolating RNC-mRNA from complex tissues and specific cell types	Stably transfected cell lines need to be established to produce labeled ribosomal proteins; over-labeling of ribosomal proteins may alter the structure and properties of the ribosome	Inada et al. (2002); Heiman et al. (2014)
Ribo-Seq	Accurately locates genes under translation; accurately quantifies gene translation levels; instantaneously measures translation efficiency; obtains ribosome position, density, ORF, and uORF information	Complex experiment; expensive; can only detect ribosome-protected RNA fragments; poor reproducibility	Ingolia et al. (2009); Ingolia et al. (2019)

Ribo-seq is based on high-throughput sequencing to detect RNA translation at the whole genome level. This technique is based on the following strategies: 1) degradation of ribosome-free RNA fragments and ribosome-nascent peptide chain complexes with low concentrations of RNase; 2) removal of ribosomes; 3) detection of small fragments (26–34 bp in length) of RNA undergoing translation whilst protected by ribosomes using second-generation sequencing technology (Ingolia et al., 2012; Ingolia et al., 2019). These ribosome-protected RNA fragments are termed ribosome footprints (RFs), which reveal the location and density of the ribosome during the translation of RNA fragments (Ingolia, 2016). Although Ribo-seq enables the detection of fragments of 26–34 bp in length undergoing translation, it usually generates 20–30 GB of data, which might represent nearly the entirety of translated sequences of an organism, thus predicting translation more accurately (Ingolia et al., 2019). Taken together, Ribo-seq has several advantages such as precise localization of genes being translated, accurate quantification of translation levels, and transient measurement of translation efficiency. In addition, compared with conventional RNC-seq, Ribo-seq enables a more accurate prediction of translated protein abundance, thus yielding more reliable results, with a lower rate of false positives.

Ribo-seq can help to unravel translational mechanisms when combined with RNA-seq, small RNA-seq, m6A-seq, single-cell RNA (ScRNA)-seq, and other sequencing methods (Calviello and Ohler, 2017; La Manno, 2019; Zong et al., 2021). Thus, in the study of lncRNAs with coding ability with the aim to unravel the greatest potential for association with certain species or diseases, it is recommended to combine Ribo-seq with RNA-seq or lncRNA-seq (Yan et al., 2021). On this basis, new micropeptides encoded by lncRNAs can be further explored and validated by combined analysis with peptidomics (Zhang et al., 2014; Vitorino et al., 2021). Peptidomics comprises the study of endogenous micropeptides or small proteins in organisms and/or compartments (cells, tissues, body fluids), being generally considered proteomics of molecules of low molecular weight (Baggerman et al., 2004). Using peptidomics it is possible to effectively enrich endogenous peptides of low molecular weight and/or low abundance, thus enabling their identification by liquid chromatography-tandem mass spectrometry (LC-MS/MS), hence a more accurate micropeptide functional annotation and differential database construction (Fabre et al., 2021). Therefore, Ribo-seq can be combined with RNA-seq or lncRNA-seq and peptidomics to obtain the most comprehensive characterization of potentially translated lncRNAs. Furthermore, considering the existence of translational regulation, correlation between transcriptome and proteome data tends to be low (Kumar et al., 2016). Thus, quantification at the translation level creates the possibility of establishing a better correlation between multi-omics data and an in-depth study of the mechanisms underlying translational regulation. Collectively, Ribo-seq can be considered an important method for the study of lncRNAs coding ability, which, when combined with multi-omics analysis, constitutes an important strategy to further validate obtained data and explore the functions of novel micropeptides encoded by lncRNAs.

### 3.2 Application of Bioinformatics to Predict the Coding Potential of lncRNAs

With the advent of high-throughput sequencing technologies, several lncRNA transcripts with coding potential have been found in different organisms. However, identification, prediction, and characterization of lncRNAs with coding ability can be challenging. Therefore, a wide variety of computational tools, software, and databases have been created for predicting and distinguishing non-coding and coding transcripts, among which can be cited sORF finder (Hanada et al., 2010), PhyloCSF (Lin et al., 2011), CNCI (Sun K et al., 2013), CPC2 (Kang et al., 2017), and CNIT (Guo et al., 2019).

Coding Potential Calculator (CPC) is a widely used method for assessing the coding potential of transcripts based on sequence features and the use of vector machines. CPC can distinguish coding and non-coding transcripts with high accuracy, but it requires sequence-to-sequence comparisons which relatively delays the analysis (Kong et al., 2007). The upgraded version CPC2 was released in 2017, which contains an accurate coding potential calculator which assesses the intrinsic features of transcript sequences, allowing for a faster and more reliable assessment of RNA coding potential (Kang et al., 2017). In addition, CPC2 is species-neutral, being thus applicable to the analysis of transcriptome data of non-model organisms (Kang et al., 2017). Furthermore, CPC2 is one of the latest lncRNA identification tools released, thus representing a considerable advancement in lncRNA coding potential identification.

In addition, predicting potential sORF in lncRNAs using bioinformatics or software is a current research trend. The ORF Finder analysis tool has been widely used and can predict all possible sORFs of lncRNAs with the corresponding amino acid sequences (Sayers et al., 2021). Subsequently, the deduced amino acid sequence can be queried against the Pfam (Mistry et al., 2021) and conserved domain database (CDD) (Lu et al., 2020) to further confirm the predicted sORFs.

In addition, conserved sequences of the coding region of lncRNAs can be determined by a variety of tools, e.g., PhyloCSF (Lin et al., 2011), RNAcode (Washietl et al., 2011), among others. A large proportion of lncRNA-encoded micropeptides are associated with intracellular membrane structures (Pang et al., 2020). The transmembrane segment of micropeptides can be predicted using the tools TMHMM or TMpred to determine the localization of the target micropeptide in the cell (intracellular, transmembrane or extracellular) (Krogh et al., 2001; Duvaud et al., 2021). Signal peptide prediction of transmembrane micropeptides can be conducted in SignalP further helped the researchers to predict the mode of action of micropeptides (Petersen et al., 2011; Almagro Armenteros et al., 2019). Subsequently, hydropathicity or hydrophobicity mapping of micropeptides is performed using ProtScale in the Expasy Bioinformatics Resource database (Duvaud et al., 2021), which in turn provides a reference for the identification of micropeptide transmembrane regions. In addition, the SWISS-MODEL in the Expasy database can be applied to homology modelling of protein structures and complexes to generate reliable protein models (Waterhouse et al., 2018), which can enable an in-depth analysis of the biological functions and structural features of lncRNA-encoded micropeptides. These bioinformatics prediction tools have been widely used; however, there are several other databases and computational tools to predict protein structure and lncRNAs coding potential which have not been mentioned herein and still require further validation by the research community.

It is known that RNAs can be classified based on their protein-coding ability into ncRNA and mRNA. However, with research advancements, an increasing number of ncRNAs with coding functions and mRNAs with non-coding functions have been described, which contrasts previous knowledge of RNA classification and function. Simultaneously, the emergence of bifunctional RNAs has stretched the boundaries between coding and non-coding RNAs and prompted researchers to reconsider the specific roles and the underlying mechanisms of RNAs in function and evolution (Nam et al., 2016). This suggests that bifunctional RNAs, i.e., those with coding and non-coding functions (cncRNA), may be worth exploring further (Huang et al., 2021). In 2020, Huang et al. (2021) established a cncRNAdb database following a comprehensive characterization of cncRNA; the current version of this database contains approximately 2,600 functional entries with experimental evidence of cncRNAs, comprising over 2,000 RNAs found in more than twenty species (including over 1,300 translated ncRNAs and over 600 untranslated mRNAs). This database can be used to further elucidate the functions and mechanisms of cncRNA, thus providing a valuable resource for future studies. Other databases also allow annotation of coding-capable lncRNAs, e.g., LNCipedia (Volders et al., 2019), lnCAR (Zheng et al., 2019), among others. All relevant computational tools, software and databases cited herein are summarized in Tables 2–4.

**TABLE 2:** ORF prediction and evaluation related calculation tools.

Name	Characteristics	Website	References
CPC	Use sequence features and support vector machines (SVM) to evaluate the protein coding potential of transcripts; assessing the scope, quality, integrity of ORFs	http://cpc.cbi.pku.edu.cn	Kong et al. (2007)
sORF finder	Package for identifying sORF with high encoding potential	http://evolver.psc.riken.jp/	Hanada et al. (2010)
PhyloCSF	Based on the formal statistical comparison of phylogenetic codon models, the nucleotide sequence alignment of multiple species is analyzed to determine whether it may represent a conserved protein coding region; it can delimit likely protein-coding ORFs within transcript models that include untranslated regions	http://compbio.mit.edu/PhyloCSF	Lin et al. (2011)
RNAcode	Comparison of conserved regions in coding and non-coding regions in sequence data and evaluation of coding potential; analysis of sORF or bifunctional RNAs	http://wash.github.com/rnacode	Washietl et al. (2011)
CNCI	Classification of protein-coding and long non-coding transcripts using sequence intrinsic composition (adjacent nucleotide triplets) (SVM-based)	http://www.bioinfo.org/software/cnci	Sun L et al. (2013)
CPAT	The coding potential assessment tool uses a permutation-free logistic regression model that can ORFs size and coverage to be assessed	http://code.google.com/p/cpat/	Wang T et al. (2013)
iSeeRNA	Identification of long intergenic non-coding RNA (lincRNA) transcripts in transcriptome sequencing data (SVM-based)	http://www.myogenesisdb.org/iSeeRNA	Sun K et al. (2013)
PLEK	Efficient alignment-free computational tool for differentiating coding and non-coding transcripts in RNA-seq transcriptomes of species lacking a reference genome (SVM-based)	https://sourceforge.net/projects/plek/files/	Li et al. (2014)
LncRNA-ID	The tool calculates the coding potential of transcripts based on a machine learning model (random forest) and multiple features	https://github.com/zhangy72/LncRNA-ID	Achawanantakun et al. (2015)
lncRNA-MFDL	By fusing multiple features and using deep learning classification algorithms to identify human lncRNA, coding and long non-coding RNA can be quickly distinguished	http://compgenomics.utsa.edu/lncRNA_MDFL/	Fan and Zhang, (2015)
COME	A multi-feature-based coding potential calculation tool for lncRNA coding potential assessment	https://github.com/lulab/COME	Hu et al. (2017)
CPC2	A fast and accurate coding potential calculator based on intrinsic sequence features for ORF feature evaluation (SVM-based)	http://cpc2.cbi.pku.edu.cn	Kang et al. (2017)
CNIT	A tool for identifying protein coding and long non-coding transcripts based on intrinsic sequence composition (upgraded version of CNCI)	http://cnit.noncode.org/CNIT	Guo et al. (2019)
ORF Finder	A software provided by NCBI that performs six-frame translation of a nucleotide sequence, allowing all possible ORFs to be inferred	https://www.ncbi.nlm.nih.gov/orffinder/	Sayers et al. (2021)

**TABLE 3:** Micropeptide information and structure-related prediction tools.

Name	Characteristics	Website	References
TMHMM	Prediction software for transmembrane structural domains (using hidden Markov model to predict the topological structure of transmembrane proteins)	http://www.cbs.dtu.dk/services/TMHMM/	Krogh et al. (2001)
TMpred	Predict the transmembrane regions and directions	https://embnet.vital-it.ch/software/TMPRED_form.html	Duvaud et al. (2021)
SignalP	Signal peptide prediction tool	http://www.cbs.dtu.dk/services/SignalP/	Almagro Armenteros et al. (2019)
ProtScale	An online tool for mapping the hydrophilic and hydrophobic atlas of proteins	https://web.expasy.org/protscale/	Duvaud et al. (2021)
SWISS-MODEL	An automated protein structure homology modeling platform that uses comparative methods to generate protein 3D models	https://swissmodel.expasy.org	Waterhouse et al. (2018)
I-TASSER	An integrated platform for automated protein structure and function prediction based on the sequence- to-structure-to-function paradigm	https://zhanglab.ccmb.med.umich.edu/C-I-TASSER/2019-nCov/	Yang et al. (2015)
AlphaFold2	A tool for accurately predicting the 3D structure of a protein based on its amino acid sequence	https://github.com/deepmind/alphafold	Jumper et al. (2021)
RoseTTAFold	A tool for accurate structure prediction of proteins and protein complexes using three-track neural networks	https://github.com/RosettaCommons/RoseTTAFold	Baek et al. (2021)

**TABLE 4:** Commonly used databases for micropeptide research.

Name	Characteristics	Website	References
BLAST	A tool for similarity analysis in protein databases or gene databases to find sequences that are similar to the query sequence. This includes patterns such as blastp, blastx, etc	https://blast.ncbi.nlm.nih.gov/Blast.cgi	Sayers et al. (2021)
Pfam	A database that classifies protein sequences into families and domains, which can be queried for protein conserved structural domains	http://pfam.xfam.org/	Mistry et al. (2021)
CDD	NCBI conserved domain database, annotated biomolecular sequences with evolutionarily conserved protein domain footprint positions, as well as functional sites deduced from these footprints	https://www.ncbi.nlm.nih.gov/Structure/cdd/cdd.shtml	Lu et al. (2020)
cncRNADB	Manually manage a resource database of bifunctional RNA (cncRNA) with protein-coding and non-coding functions	http://www.rna-society.org/cncrnadb/	Huang et al. (2021)
LNCipedia	A public database for storing lncRNA sequences and annotation information	https://lncipedia.org/	Volders et al. (2019)
lnCAR	A comprehensive resource for lncRNA from cancer arrays (including lncRNA coding information)	https://lncar.renlab.org/	Zheng et al. (2019)
NONCODE	A database annotated with a large amount of lncRNA information	http://www.noncode.org/	Zhao et al. (2016)
UCSC	Genome Browser database that provides high quality visualization of genomic data and genome annotation. Has tools such as BLAT, track hubs, etc. for viewing, analyzing and downloading data	https://genome.ucsc.edu	Navarro Gonzalez et al. (2021)
UniProt	The most comprehensive database of protein sequence and annotation information, consisting of UniProtKB, UniRef, and UniParc, and integrating data from three major databases, swiss-prot, TrEMBL, and PIR-PSD	https://www.uniprot.org/	UniProt, (2021)
Expasy	A database of reliable and most advanced bioinformatics service tools and resources is stored. Has tools such as protscale, TMpred, etc. for viewing, analyzing, and downloading data	https://www.expasy.org/	Duvaud et al. (2021)
LncPep	The lncRNA coding peptides database	http://www.shenglilabs.com/LncPep/	Liu et al. (2022)
SPENCER	A comprehensive database for small peptides encoded by noncoding RNAs in cancer patients	http://spencer.renlab.org	Luo et al. (2022)

### 3.3 Experimental Identification of lncRNAs Coding Potential

Through combined multi-omics analysis and bioinformatics prediction, several lncRNAs with promising application in research and coding potential have been described. After prediction, these lncRNAs require experimental identification. Firstly, RNA-fluorescence *in situ* hybridization (RNA-FISH) technology is used to determine lncRNA localization in the cell; since translation of micropeptides mostly occurs in the cytoplasm, determining their localization improves inferring their potential function of lncRNA-encoded micropeptides (Huang et al., 2017; Yan et al., 2021). A FLAG/HA-tag system was cloned before the stop codon of the potential sORF of this lncRNA, and the fusion sequence containing the FLAG/HA-tag was cloned into a plasmid vector for *in vitro* cell transfection (Pang et al., 2020); after transfection into target cell line or wild-type cells, the relative expression of the micropeptide was detected by western blotting and immunofluorescence assays using anti-FLAG/HA tag antibodies (Wu S et al., 2020). Alternatively, sORFs of lncRNAs can be fused to the N-terminal end of green fluorescent protein (GFP) vectors with mutated start codons, and the relative expression of micropeptides can be detected by western blotting and immunofluorescence assays with anti-GFP antibodies (Zhu et al., 2020). Immunoprecipitation (Co-IP) in tandem with mass spectrometry (MS) analysis of ORF-GFP fusion peptides can be performed using anti-GFP antibodies to further identify lncRNA-translated micropeptides (Wang L et al., 2020). However, since most GFP-tags are larger in size than lncRNA-encoded micropeptides, and GFP-tagged micropeptides may alter the phenotype of micropeptides, FLAG-tag fused constructs are mostly used in experimental identification of lncRNAs coding potential. In addition, the CRISPR-Cas9 system can be used to knock in FLAG-tags before the stop codon of the lncRNAs locus in target cells, and the relative expression of the resulting micropeptides can be determined using by Western blotting and immunofluorescence with anti-FLAG antibodies, thus validating the coding ability of lncRNAs (Anderson et al., 2015; Wang Y et al., 2020).

Determining the endogenous expression of micropeptides is important to infer whether micropeptides play a regulatory role in the organism. The verification of micropeptide endogenous expression can be performed using the following techniques: 1) designing polyclonal antibodies based on the micropeptide, and further confirmation of micropeptide production using western blotting on target fresh tissues or cells; 2) using MS analysis to obtain the fingerprint of the target micropeptide, which can be then discovered by comparison; 3) blocking cell translation using actinomycin (CHX) or antimicropeptide antisense oligonucleotides (OMA), followed by detection of micropeptide expression over time (Walther and Mann, 2010; Li et al., 2017; Guo et al., 2020; Li et al., 2021). In addition, several micropeptides encoded by lncRNAs have been described to be associated with intracellular membrane structures (Pirkmajer et al., 2017; Pang et al., 2020). To determine whether micropeptides are associated with cell membrane structures, in addition to the bioinformatics analysis discussed above, experimental validation is further necessary, which may include the following: 1) extraction of membrane and cytoplasmic proteins from cells followed by western blotting detection using polyclonal antibodies targeting the micropeptide; 2) imaging flow cytometry techniques (Han et al., 2016; Mikami et al., 2020; Pang et al., 2020). In addition, it has been speculated that micropeptides can act as components of structural proteins and signaling molecules, which require further demonstration.

**FIGURE 1:**
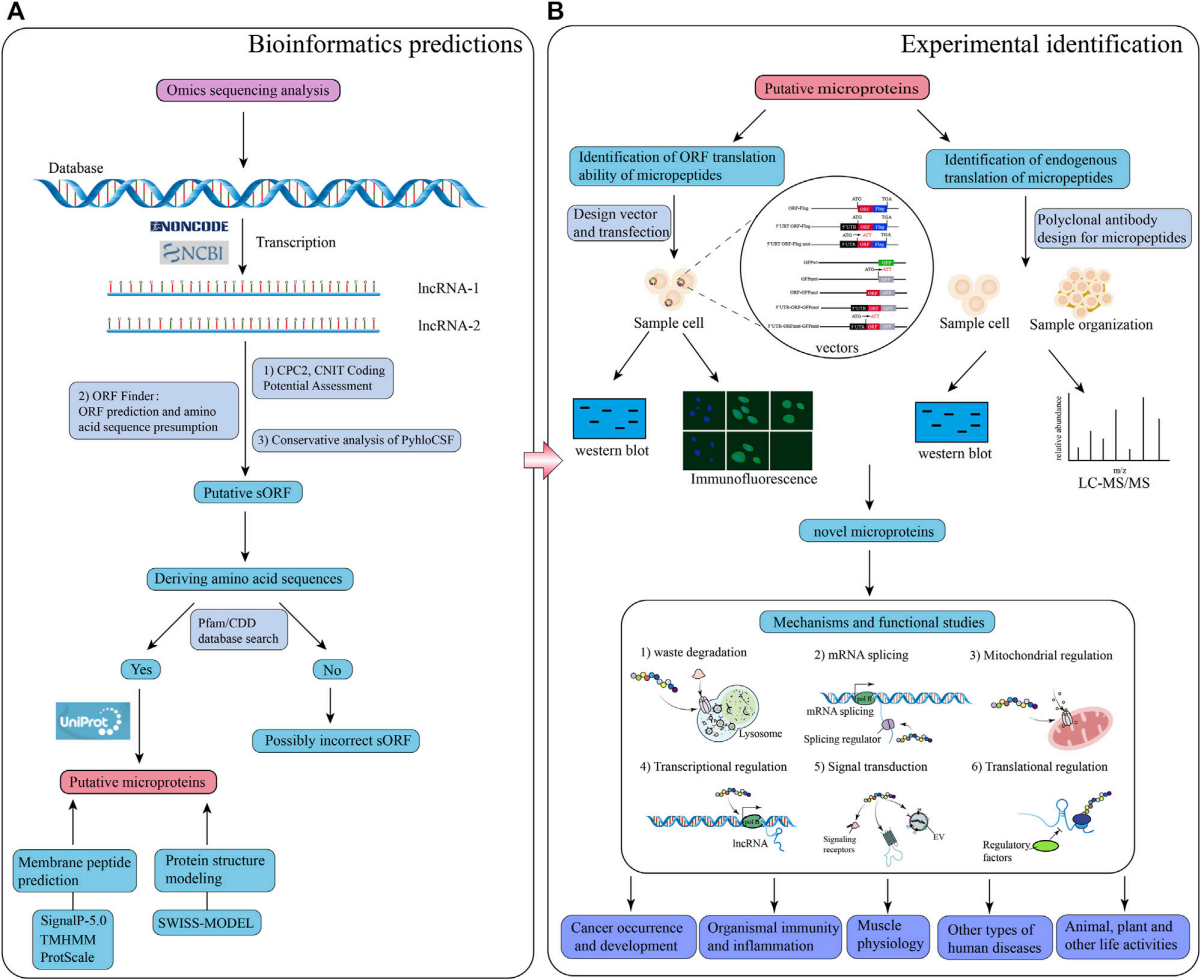
Schematic illustration of the workflow for bioinformatics prediction and experimental analysis of lncRNA-encoded micropeptides. **(A)** Bioinformatics prediction: firstly, construct a database of putative lncRNA-encoding micropeptides by applying the results of omics sequencing, and search the putative lncRNA sequences with coding potential through NCBI or NONCODE database; secondly, use calculation tools, and databases such as CPC2, CNIT, ORF Finder, PyhloCSF, etc. to evaluate the coding potential of the putative lncRNA, and deduce the corresponding sORF, and amino acid sequence; thirdly, the deduced amino acid sequences were put into the Pfam and CDD databases to look for them, and if they matched, the search for the putative micropeptide information was continued through the UniProt database; finally, the characteristics and structure of the putative micropeptide were predicted and modeled through calculation tools and databases such as SignalP-5.0, TMHMM, ProtScale and SWISS-MODEL; **(B)** Laboratory identification: design a series of special vectors to be transfected into specific cells, and apply western blot and immunofluorescence experiments to identify micropeptides; meanwhile, polyclonal antibodies to this micropeptide were designed, and detected by western blot and LC-MS/MS experiments on sample cells and tissues. Based on the results of both experimental procedures, the putative micropeptide was identified as a novel micropeptide, and then the function and mechanism of the micropeptide were investigated.

Previous studies have revealed that lncRNAs associated with ribosomes do not necessarily encode micropeptides; furthermore, if they are coding lncRNAs, encoded micropeptides might still lack functionality. In addition, certain lncRNAs exert their regulatory effects directly rather than through their encoded micropeptides (Gaertner et al., 2020). Therefore, it is necessary to verify whether lncRNAs are inherently functional or only through their encoded micropeptides. It has also been found in earlier studies that, although most micropeptides encoded by lncRNAs may be nonfunctional and highly unstable, about 9% of lncRNA-encoded peptides are conserved in the ORFs of mice transcripts (Ji et al., 2015). Therefore, functional validation of micropeptides encoded by lncRNAs is required to confirm their functionality. Special vectors of lncRNA (knockdown or overexpression) can be designed to transfect cells to enable the impact of introduced vectors in cell fate. In addition, rescue experiments can be conducted to verify whether the lncRNA itself or the encoded micropeptide is responsible for the regulation. After demonstrating the function of the encoded micropeptide, mice models can be used to validate micropeptide activity and regulatory effect *in vivo* (Zhu et al., 2020). These newly discovered functions of lncRNA-encoded micropeptides have greatly enriched the current understanding of lncRNAs. However, due to technological challenges and difficulties in synthesizing polyclonal antibodies for micropeptides, there are still relatively few studies in this field, being thus necessary to explore further. A suggested workflow for studying lncRNA-encoded micropeptides is shown in Figure 1.

After verifying that lncRNA-encoded micropeptides are functional micropeptides, the potential regulatory mechanisms behind these micropeptides have become a pressing issue for subsequent research. CO-IP and MS analysis were applied to find proteins interacting with the micropeptides (Li et al., 2021); RNA-Seq of cells knocked down for micropeptides to look for differential genes and associated signalling pathways (Pang et al., 2020); the JASPAP (the open-access database of transcription factor binding profiles) was used to find the transcription factor that binds to the micropeptide, and dual-luciferase reporter gene vector and chromosomal immunoprecipitation (CHIP) assay were designed to verify the transcription factor that binds to the micropeptide (Castro-Mondragon et al., 2022).

## 4 Potential Regulatory Roles of lncRNA-Encoded Micropeptides

With the increasing knowledge of lncRNAs encoding micropeptides, the potential regulatory mechanisms of these molecules have also been receiving increasing attention. This suggests that certain mechanisms believed to be regulated by lncRNAs might not be related to an inherent function of lncRNAs but to the micropeptides they encode. This new piece of evidence may override previous knowledge about lncRNAs, suggesting that this phenomenon should be more carefully explored to enable the discovery of appropriate regulatory factors. This will also provide more reliable information for disease and cancer treatment as well as for improving plant and animal productivity.

In 2014, Slavoff et al. (2014) identified the sORF-encoded micropeptide SEP in humans which was shown to stimulate DNA double-strand-break junctions by non-homologous end joining and be involved in DNA repair. In addition, the bifunctional gene lncRNA-Six1, located 432 bp upstream of the gene encoding the protein six homology frame 1 (Six1), was shown to cis-regulate the *Six1* gene encoding the protein; the micropeptide encoded by this lncRNA was also shown to activate the *Six1* gene, which has been shown to be associated with DNA repair (Cai et al., 2017). This indicates that lncRNA-encoded micropeptides might be involved in gene expression and DNA repair processes. Another micropeptide (namely NoBody) encoded in humans in the LINC01420/LOC550643 sORF has been shown to be involved in mRNA turnover and NMD by interacting with mRNA decapping proteins to remove the 5' cap of mRNA to promote 5' to 3' decay (D'Lima et al., 2017). Moreover, NoBody was localized in mRNA decay-associated RNA-protein granules, namely P-bodies. In addition, NoBody levels were shown to be negatively correlated with the number of cellular P-bodies and alter the steady-state levels of cellular NMD substrates (D'Lima et al., 2017), which also suggests that lncRNA-encoded micropeptides might be involved in mRNA conversion and NMD. In addition, lncRNA-encoded micropeptides were shown to interact with multiple splicing regulators to influence RNA splicing (Meng et al., 2020).

Furthermore, Pang et al. (2020) identified a conserved peptide, SMIM30, encoded by LINC00998, which activates the downstream MAPK signaling pathway by driving membrane anchoring and phosphorylation of the non-receptor tyrosine kinase SRC/YES1. This reveals a novel regulatory mechanism of lncRNA-encoded peptides related to the activation of signaling pathways. In addition, lncRNA-encoded micropeptides were shown to regulate mRNA stability and expression by interacting with m6A reader-associated proteins (Zhu et al., 2020), which may provide a guidance for future studies. However, whether these transcriptional modifications have regulatory effects on lncRNA-encoded micropeptides remains to be further explored.

## 5 Biological Functions of lncRNA-Encoded Micropeptides

### 5.1 Micropeptides Associated With Skeletal Muscle Development

**FIGURE 2:**
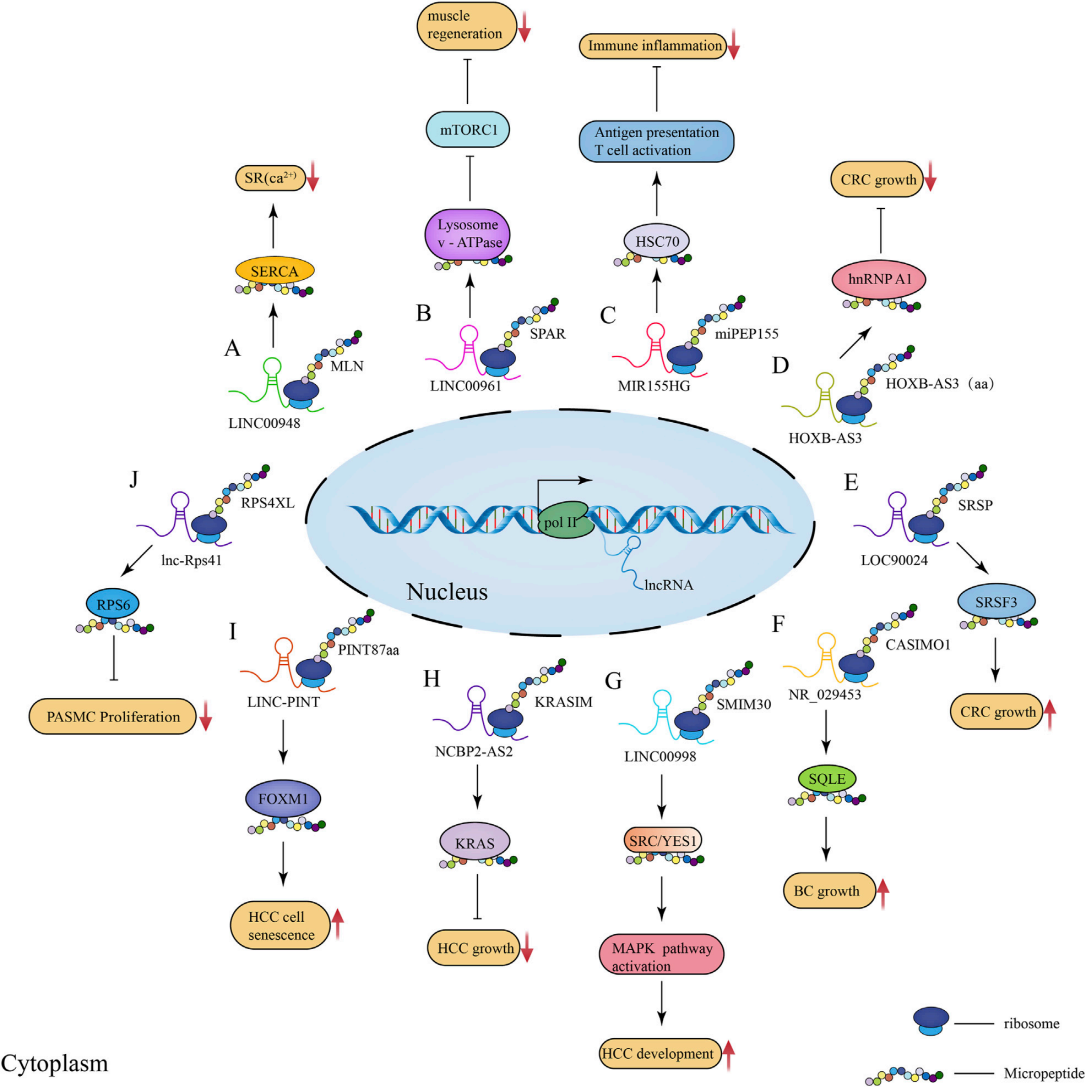
Schematic illustration of the regulatory role of lncRNA-encoded micropeptides in muscle physiological processes as well as disease and tumorigenesis and development. **(A)** Mechanism of action diagram of micropeptide MLN encoded by lncRNA LINC00948 in skeletal muscle physiological process; **(B)** Mechanism of action diagram of conserved peptide SPAR encoded by lncRNA LINC00961 in muscle regeneration process; **(C)** Mechanism of action diagram of micropeptide miPEP155 (P155) encoded by lncRNA MIR155HG in immunity and inflammation; **(D)** Mechanism of action diagram of the 53-aa conserved peptide encoded by lncRNA HOXB-AS3 in CRC; **(E)** Mechanism of action diagram of the micropeptide SRSP encoded by lncRNA LOC90024 in CRC; **(F)** Mechanism of action diagram of the micropeptide CASIMO1 encoded by lncRNA NR_029453 in BC; **(G)** Mechanism of action diagram of the conserved peptide SMIM30 encoded by LINC00998 in HCC; **(H)** Mechanism of action diagram of the 99-aa conserved peptide KRASIM encoded by lncRNA NCBP2-AS2 interacting with KRAS in HCC; **(I)** Mechanism of action diagram of the micropeptide PINT87aa encoded by LINC-PINT interacting with FOXM1 in HCC cell senescence; **(J)** Mechanism of action diagram of the micropeptide RPS4XL encoded by lnc-Rps41 interacting with RPS6 in PASMC.

Skeletal muscle is the largest and most important constitutive tissue of the human locomotor system, thus playing a crucial role in locomotion and glucolipid metabolism homeostasis (Frontera and Ochala, 2015). In 2013, Magny et al. (2013) identified two peptides shorter than 30 aa in length in *Drosophila* heart tissue, and these peptides were shown to affect muscle homeostasis by regulating calcium transport. This suggests that micropeptides may be important regulators of calcium-dependent signaling in muscle tissue. In 2015, when investigating how micropeptides regulate muscle movement, Anderson et al. (2015) found that myoregulin (MLN), encoded by a skeletal muscle-specific lncRNA, could control muscle relaxation by blocking Ca^2+^ uptake into the sarcoplasmic reticulum (SR) and interaction with cardiac SR Ca^2+^-ATPase (SERCA) (Figure 2A). Considering that SERCA plays Figure 2A an important role in the regulation of calcium homeostasis in cardiac myocytes (Anderson et al., 2015), these observations suggest that micropeptides might play an important regulatory role in skeletal muscle physiology. Subsequently, Anderson et al. (2016) further identified two additional regulatory proteins, namely endoregulin (ELN) and another-regulin (ALN) encoded by genes *1110017F19Rik/SMIM6*, and *1810037I17Rik*, which share key amino acid residues with their muscle-specific counterparts and function as direct inhibitors of SERCA pump activity. Additionally, a 34-aa-long micropeptide, DWarf Open Reading Frame (DWORF), encoded by a muscle-specific lncRNA and localized in the SR membrane, was shown to enhance SERCA activity by displacing SERCA inhibitors, phosphoproteins, myosin, and myoregulatory proteins to enhance muscle contraction (Nelson et al., 2016). These findings indicate that micropeptides act as both SERCA inhibitors and activators, thus mediating the regulation of calcium homeostasis in cardiac myocytes, and showing their importance in skeletal muscle physiology.

In addition, micropeptides can also regulate muscle regeneration by interacting with mechanistic target of rapamycin complex 1 (mTORC1). Matsumoto et al. found that SPAR, a conserved peptide encoded by LINC00961, could inhibit mTORC1 activation by interacting with lysosomal v-ATPase (Figure 2B; Matsumoto et al., 2017). Considering that activated mTORC1 promotes muscle regeneration, it can be speculated that SPAR acts as an inhibitor of muscle regeneration. Subsequently, Rion and Ruegg (2017) and Tajbakhsh (2017) further explained the mechanism underlying SPAR-mediated inhibition of mTORC1, further validating the proposed regulating mechanism of muscle regeneration. In addition, it has been proposed that lncRNA-encode micropeptides can regulate skeletal muscle movement by influencing mitochondrial metabolic processes. Makarewich et al. (2018) identified an lncRNA annotated as 1500011K16Rik and LINC00116 in mouse and human genomes, respectively, encoding a conserved peptide MOXI that binds to the mitochondrial trifunctional protein at the mitochondrial inner membrane, as well as affects the mitochondrial metabolism and energy homeostasis regulation. Knockdown of MOXI reduced the ability of cardiac and skeletal muscle mitochondria to metabolize fatty acids and significantly reduced muscle motility (Makarewich et al., 2018). Another LINC00116 found enriched in skeletal muscle and heart was shown to encode a micropeptide, Mtln, that affects muscle motility by regulating fatty acid oxidation and mitochondrial metabolic processes (Stein et al., 2018). Chugunova et al. (2019) further investigated Mtln and validated the important mechanism of action of this micropeptide in linking respiration and lipid metabolism, as well as its importance in the control of cell fate.

It is known that skeletal muscle development requires fusion of mononuclear progenitor cells to form multinucleated myotubes in a critical but poorly understood process (Hindi et al., 2013). In 2017, Zhang et al. (2017) discovered that the micropeptide Minion (fusion microprotein inducer) encoded by LOC10192972 controls cell fusion and muscle tissue formation by influencing myogenic progenitor cells to form syncytial myotubes. Moreover, it has been shown that Minion-deficient mice died perinatally and exhibited a significant reduction in fused muscle fibers (Zhang et al., 2017). This observation further validates the belief that skeletal muscle development requires the fusion of mononuclear progenitor cells to form multinucleated myotubes. Another micropeptide that has been shown to play a key role in muscle development is LEMP, encoded by the lncRNA MyolncR4, which is highly conserved in vertebrate species (Wang L et al., 2020). LEMP was shown to promote muscle formation and regeneration, and LEMP-deficient mutants had impaired muscle development (Wang Y et al., 2020). Collectively, these findings reveal that lncRNA-encoded micropeptides play an important regulatory role in muscle development, and that certain lncRNAs seemingly lacking coding ability may have been misannotated.

### 5.2 Micropeptides Related to Immune System Inflammatory Response

The latest research findings have revealed that lncRNA-encoded micropeptides play an important role in human innate immunity. In 2018, Jackson et al. (2018) identified a micropeptide encoded by lncRNA Aw112010, which was shown to be essential for the innate immune response *in vivo*, coordinating mucosal immunity under bacterial infections and colitis; moreover, this micropeptide is translated from a non-canonical ORF. Therefore, mis-annotation of genes containing non-canonical ORFs as non-coding RNAs may obscure the role of a large number of previously unidentified protein-coding genes in innate immunity and disease. Another study revealed that lncRNA 1810058I24Rik was downregulated in both human and murine myeloid cells exposed to lipopolysaccharides (LPS), as well as in other Toll-like receptor (TLR) ligands and inflammatory cytokines (Bhatta et al., 2020); this lncRNA encodes a 47-aa-long mitochondrial micropeptide-47 (Mm47) which might be involved in the immune response by activating the Nlrp3 inflammasome to monitor various pathogens and threatening signals (Mangan et al., 2018; Bhatta et al., 2020). Later, Niu et al. (2020) found that the lncRNA MIR155HG encodes the micropeptide miPEP155 (P155) which interacts with the heat shock cognate protein 70 (HSC70) to mediate antigen presentation and T cell initiation as well as suppress autoimmune inflammation (Figure 2C). Collectively, these findings reveal micropeptides as modulators of antigen presentation and inhibitors of inflammatory diseases, suggesting that micropeptides play an important role in immunity and inflammation, which could offer insights for novel treatments.

### 5.3 Micropeptides Related to Cancer Development

Cancer is a major burden of human diseases. A number of functional micropeptides have been suggested to play a key regulatory role in various human diseases, including cancer, which may constitute a valuable resource for disease and cancer treatments.

Melanoma is among the most dangerous types of skin cancer. Between 2008 and 2013, multiple antigens (e.g., MELOE-1, MELOE-2, and MELOE-3) translated from multiple sORFs of lncRNAs and multiple cis-trans RNAs were found overexpressed in melanoma cells, being also involved in T cell surveillance mechanisms (Godet et al., 2008; Carbonnelle et al., 2013; Charpentier et al., 2016); these could provide optimal T cell targets and therapeutic strategies for melanoma immunotherapy. Interestingly, Huang et al. (2017) found a 53-aa-long conserved peptide encoded by lncRNA HOXB-AS3 in colorectal cancer (CRC) cells, which could inhibit the growth of CRC cells by binding to the heterogeneous nuclear ribonucleoproteins A1 (hnRNP A1) to mediate the cancer metabolic reprogramming process (Figure 2D). Meng et al. (2020) described that the micropeptide SRSP encoded by LOC90024 interacts with serine/arginine-rich splicing factor 3 (SRSF3) to promote tumorigenesis and progression in CRC (Figure 2E). Moreover, micropeptides encoded by lncRNAs have been associated with breast cancer (BC). The micropeptide CASIMO1 translated from transcripts misannotated as lncRNA was found overexpressed in hormone receptor-positive breast tumors; when it was silenced, reduced proliferation was observed in a variety of BC cell lines (Polycarpou-Schwarz et al., 2018). Moreover, CASIMO1 was found to interact with BC oncogenic gene squalene epoxidase (*SQLE*) in the regulation of cellular lipid homeostasis and thus cancer development (Figure 2F; Polycarpou-Schwarz et al., 2018). Other lncRNA-encoded micropeptides were also found to play a key regulatory role in BC, such as lncRNA EPR-encoded micropeptide (Rossi et al., 2019), LINC00665-encoded micropeptide CIP2A-BP (Guo et al., 2020), and LINC00908-encoded 60-aa-long micropeptide ASRPS (Wang L et al., 2020). The discovery of these key micropeptides provides valuable information on potential therapeutic targets for the treatment of BC as well as clinical research.

Recently, Pang et al. (2020) described that LINC00998 encodes the conserved peptide SMIM30 which promotes hepatocellular carcinoma (HCC) tumorigenesis by regulating cell proliferation and migration (Figure 2G). In this study, a new mechanism of HCC tumorigenesis promoted by the micropeptide has been proposed, which could potentially be used as a new target for HCC therapy as well as a biomarker for HCC diagnosis and prognosis. Xu et al. (2020) identified a 99-aa-long conserved micropeptide, KRASIM, encoded by lncRNA NCBP2-AS2, which was shown to inhibit HCC oncogenic signals, cancer cell growth and proliferation (Figure 2H). These results demonstrate a novel micropeptide inhibitor and provides new insights into the regulatory mechanisms of oncogenic signaling and HCC therapy. Moreover, when exploring the mechanisms of micropeptide function in HCC cell senescence, Xiang et al. (2021) found that the micropeptide PINT87aa, encoded by LINC-PINT, could function as a biomarker and a key regulator of HCC cell senescence, being thus considered a potential therapeutic target for HCC (Figure 2I). In addition, it has been demonstrated that the second exon in LINC-PINT RNA can self-loop to form a circular molecule (circPINT) which encodes micropeptides and was involved in the inhibition of glioblastoma cell proliferation (Zhang et al., 2018).These interesting findings reveal that lncRNAs can self-loop and still regulate cancer progression by encoding micropeptides after self-looping, which may provide new insights for cancer and disease treatments. More recently, Cai et al. (2021) identified a micropeptide encoded by lncRNA that is abundantly present in extracellular vesicles (EVs) of glioma cancer cells, which may suggest that EVs-mediated micropeptide transfer represents a novel mechanism of intercellular communication that could potentially be applied in the diagnosis of glioma. In addition, it has been suggested that lncRNAs can encode micropeptides that form oligomers that interfere with water or ion regulation, and abnormalities in water and ion channels play an important role in cancer cell proliferation, migration, apoptosis, and differentiation (Cao et al., 2021). For instance, Cao et al. (2021) found that lncRNA DLEU1 encoding a small transmembrane peptide in glioma cells forms a pentameric channel that acts as a water channel in these cells. Furthermore, lncRNA-encoded micropeptides play an important role in other types of cancers, such as lung cancer (Lu et al., 2019) and esophageal squamous cell carcinoma (Wu P et al., 2020). Collectively, the role of lncRNA-encoded micropeptides in cancer is still poorly understood, and many regulatory mechanisms have not yet been described. Current studies have revealed that micropeptides encoded by lncRNAs, which were previously misannotated as non-coding RNAs, play an important role in cancer development and progression. However, the functions of these functional micropeptides in tumorigenesis are still poorly understood due to the limitations of current available technology for the study of lncRNAs and deserve further investigation. Moreover, the discovery of these functional micropeptides may represent a novel strategy for clinical treatment and prognosis of cancer.

### 5.4 Other Diseases

Pulmonary hypertension (PH) is a rare and fatal disease. An important pathological process in PH is related to the proliferation of pulmonary artery smooth muscle cells (PASMCs) caused by hypoxia (Hu et al., 2021). In a previous study, it was found that lnc-Rps41 with high coding capacity mediates the proliferation of PASMCs under hypoxic conditions (Liu et al., 2020); its encoded micropeptide, RPS4XL, was shown to inhibit PASMCs proliferation and reduce PH death induced by PASMCs proliferation, which could provide a potential target for early diagnosis of PH (Figure 2J; Li et al., 2021).

Myocardial infarction is a severe disease in which an acute blockage of the coronary artery occurs, causing ischemic necrosis of part of the myocardium (Piamsiri et al., 2021). Spencer et al. identified that the micropeptide SPAAR encoded by LINC00961 plays an important role in angiogenesis (Spencer et al., 2020). In addition, loss of the LINC00961/SPAAR locus was found to affect development, myocardial dynamics, and myocardial infarction cardiac response in mice (Spiroski et al., 2021), which suggests that LINC00961/SPAAR contributes to growth and development as well as basal cardiovascular function in adulthood, thus mitigating the risk of myocardial infarction. Therefore, these observations may provide a novel scientific basis and strategy for clinical treatment of cardiovascular diseases.

## 6 Conclusion and Future Perspectives

Current research on micropeptides encoded by lncRNAs has been received increasing attention. Many computational tools, software, and databases for assessing and predicting lncRNA coding potential have been developed. Moreover, several servers for micropeptide information, and structure prediction are available, which contributes to the study of micropeptides in a more systematic and simplified manner, thus provides a solid foundation for micropeptide research. Moreover, the combined analysis of data obtained by omics techniques (transcriptomics, translatomics, proteomics) constitutes a more comprehensive strategy to the analysis of processes in biological systems and to explain the complexity and the overall nature of such processes. Therefore, the progress of the field of lncRNA-encoded micropeptide research chiefly relies on establishing more systematic investigation and robust analytical tools.

Micropeptides encoded by lncRNA may be the missing part in several molecular regulatory mechanisms. Most micropeptides can regulate biological processes independently from lncRNAs and play important roles in the organism. In addition, many lncRNAs were shown to influence several disease-causing and life-sustaining processes in plants and animals; however, it remains to be elucidated whether the function of lncRNAs is related to a certain aspect of their nature or to the micropeptides they encode. Moreover, annotations in current databases of lncRNA-encoded micropeptides are available only for a few species, including human, mouse, rat, zebrafish, fly, yeast, *Caenorhabditis elegans*, *Escherichia coli*, and others. There is still a large number of species for which the lncRNA coding potential has not yet been annotated. This requires further exploration, as to enrich species database information, thus laying a solid foundation for future research.

In addition, although many lncRNAs with coding potential have been characterized, screening methods of functional micropeptides are still controversial. Considering that micropeptide screening criteria are strict and annotation is mainly based on phylogenetic conservatism analysis, a large number of non-standard translated micropeptides might have gone unnoticed, thus limiting the development of micropeptide-based application. A more in-depth study of lncRNAs and their encoded micropeptides will significantly expand the progress of research in the life sciences and provide new insights and strategies into solving the most urgent problems of the field.
